# Therapeutics of the Throat: Ignatia

**Published:** 1888-01

**Authors:** George H. Clark

**Affiliations:** Germantown, Phila.


### THERAPEUTICS OF THROAT: IGNATIA.

### OBJECTIVE.

Disease begins on right side (Lycop.).
Tonsils inflamed, hard, swollen, with small ulcers.
Increased turgescence of tonsils.
Redness, inflammation, and soreness of inner mouth.
Whole inner surface of mouth sore.
Tendency to swelling of cervical glands.

SUBJECTIVE.

Sensation as if palate were swollen, or covered with tenacious mucus.

*Sticking in palate, extending to inner ear.*

Stitches in soft palate, extending to the ear.

Sensation in palate as if sore.

Constrictive sensation in pit of throat, causing cough, as from fumes of sulphur.

*Retching (constrictive) sensation in middle of throat, as if there were a large morsel of food or a plug sticking there,* WORSE WHEN NOT SWALLOWING THAN WHEN SWALLOWING.

Pressure in throat.

Sticking low down in throat when swallowing, *it disappears on continuing to swallow, and returns when not swallowing.*

*When swallowing, it seems as though one swallowed over a bone, causing cracking.*

*Sensation as though a plug were sticking in throat, noticed when not swallowing.*

Needle-like stitches low down in throat, in quick succession, when not swallowing.

Sore throat; the internal throat is painful, as if raw and sore.

Pain in throat as from soreness, only noticed when swallowing.

Aching in throat.

*Sore throat, stitches which are not felt when swallowing.*

Sore throat, like a lump in throat, which is painfully sore when swallowing.

STICKING IN THROAT WHEN NOT SWALLOWING, AND EVEN SOMEWHAT WHILE SWALLOWING; THE MORE HE SWALLOWS, HOWEVER, THE MORE IT DISAPPEARS: IF HE SWALLOWS ANYTHING SOLID, LIKE BREAD, IT SEEMS AS THOUGH THE STICKING ENTIRELY DISAPPEARS.

Throat worse when not swallowing and when swallowing liquids; better when swallowing food.

Choking sensation from the stomach up into the throat.

Pain in throat when touched as if glands were swollen.

FORMICATION IN THE ŒSOPHAGUS.

Tearing pain in larynx, much aggravated when swallowing and breathing.

*Crawling in pharynx.*

On eating there was some difficulty in swallowing food and drink.

*He was unable to swallow bread, it seemed too dry.*

Food seems to reach as far up as the throat in the evening before falling asleep and in the morning.

Pain in glands below angle of lower jaw on motion of throat.

Sub-maxillary glands painful after walking in open air.

Drawing pain in sub-maxillary glands, which extends into jaws, after which the glands become swollen.

*Pressive pains in cervical glands.*

Pain at first pressive, afterward drawing, in sub-maxillary glands.

Sticking in one side of throat and in parotid gland when swallowing.

Aggravation—Walking in open air; sub-maxillary glands painful.
Breathing: Tearing in larynx.
Coughing: " "
Swallowing: " "
When not swallowing: Constrictive sensation in middle of throat.
Sticking in throat.
Plug in throat.
Stitches in throat.
Sticking in parotid gland.
From tobacco: The symptoms in general.

Amelioration—When swallowing: STITCHES IN THROAT.
STICKING IN THROAT.

Other remedies, having a sensation of plug in throat when not swallowing, are: *Nat. mur.* and Sulph.

Capsicum has burning in throat, worse between the acts of swallowing.

Hahnemann says of Ignatia: "It is suitable for but few cases of chronic disease, and then only with the intermediate employment of some other suitable medicine of more persistent action.

"Ignatia is not suitable for persons or patients in whom anger, eagerness, or violence is predominant, but for those who are subject to rapid alternations of gayety and disposition to weep."

It is especially suitable to nervous and hysterical females of mild but easily excited nature, and finds a prominent place where symptoms arise from or are aggravated by fright and grief.

GEORGE H. CLARK.

GERMANTOWN, PHILA.

